# The prognostic significance of human ovarian aging-related signature in breast cancer after surgery: A multicohort study

**DOI:** 10.3389/fimmu.2023.1139797

**Published:** 2023-03-07

**Authors:** Xin Hua, Qi-Wei Zhu, Yi-Nuan Zhang, Lu Cao, Meng-Di Wang, Yun-Sheng Gao, Jia-Yi Chen

**Affiliations:** ^1^ Department of Radiation Oncology, Shanghai Jiao Tong University Medical School Affiliated Ruijin Hospital, Shanghai, China; ^2^ Department of Radiation Oncology, Affiliated Tumor Hospital of Nantong University, Nantong, Jiangsu, China; ^3^ Department of Radiation Oncology, The First People’s Hospital of Foshan, Foshan, Guangdong, China

**Keywords:** ovarian ageing, breast cancer, prognosis, drug sensitivity, immune infiltration

## Abstract

**Background:**

Recent studies have shown that ovarian aging is strongly associated with the risk of breast cancer, however, its prognostic impact on breast cancer is not yet fully understood. In this study, we performed a multicohort genetic analysis to explore its prognostic value and biological features in breast cancer.

**Methods:**

The gene expression and clinicopathological data of 3366 patients from the The Cancer Genome Atlas (TCGA) cohort, the Molecular Taxonomy of Breast Cancer International Consortium (METABRIC) cohort and the GSE86166 cohort were analyzed. A total of 290 ovarian aging-related genes (OARGs) were included in the establishment of the prognostic model. Furthermore, functional mechanisms analysis, drug sensitivity, and immune cell infiltration were investigated using bioinformatic methods.

**Results:**

An eight OARG-based signature was established and validated using independent cohorts. Two risk subgroups of patients with distinct survival outcomes were identified by the OARG-based signature. A nomogram with good predictive performance was developed by integrating the OARG risk score with clinicopathological factors. Moreover, the OARG-based signature was correlated with DNA damage repair, immune cell signaling pathways, and immunomodulatory functions. The patients in the low-risk subgroup were found to be sensitive to traditional chemotherapeutic, endocrine, and targeted agents (doxorubicin, tamoxifen, lapatinib, etc.) and some novel targeted drugs (sunitinib, pazopanib, etc.). Moreover, patients in the low-risk subgroup may be more susceptible to immune escape and therefore respond less effectively to immunotherapy.

**Conclusions:**

In this study, we proposed a comprehensive analytical method for breast cancer assessment based on OARG expression patterns, which could precisely predict clinical outcomes and drug sensitivity of breast cancer patients.

## Introduction

Breast cancer is a hormone-sensitive tumor and its development and progression are closely related to the host’s hormone levels (1, 2). The decline in ovarian function, known as ovarian aging, results from a decrease in the quantity and quality of oocytes and is one of the key intrinsic determinants of hormonal changes (3). Numerous studies have shown that ovarian aging is strongly associated with the risk of breast cancer, but its prognostic impact on breast cancer is not yet fully understood. Therefore, it is of great significance to explore the prognostic implications of ovarian aging and its potential as an alternative individual therapeutic target for breast cancer.

Menarche and menopause mark the origin and end points in the process of ovarian ageing, as well as affect breast cancer risk. It has been well-documented that women who experienced menarche at an early age have an exponentially increased risk of developing breast cancer (4–7). Large cohort studies have also demonstrated that breast cancer incidence decreases with an earlier onset of menopause (8–10). Ovarian aging is a complex process with multi-linked genetic, etiological, or influencing factors and its molecular mechanisms remains largely unelucidated (3, 11). Fortunately, a new study in *Nature* conducted a large-scale genome-wide association study of ovarian ageing and identifies 290 genetic determinants of ovarian aging (12). Therefore, a comprehensive understanding of the relationship between the expression of the 290 ovarian aging-related genes (OARGs) and survival outcomes in breast cancer, would be important in determining the effects of ovarian aging in breast cancer.

Herein, this study was conducted to evaluate the prognostic profiles of OARGs in breast cancer. A novel ovarian aging-based signature for evaluating breast cancer prognosis was developed and validated in multiple cohorts. Furthermore, the present study aimed to present the prognostic landscape of OARGs in breast cancer, and screen for survival-related OARGs as biomarker candidates and potential therapeutic targets.

## Methods

### Data collection

**Figure 1:**
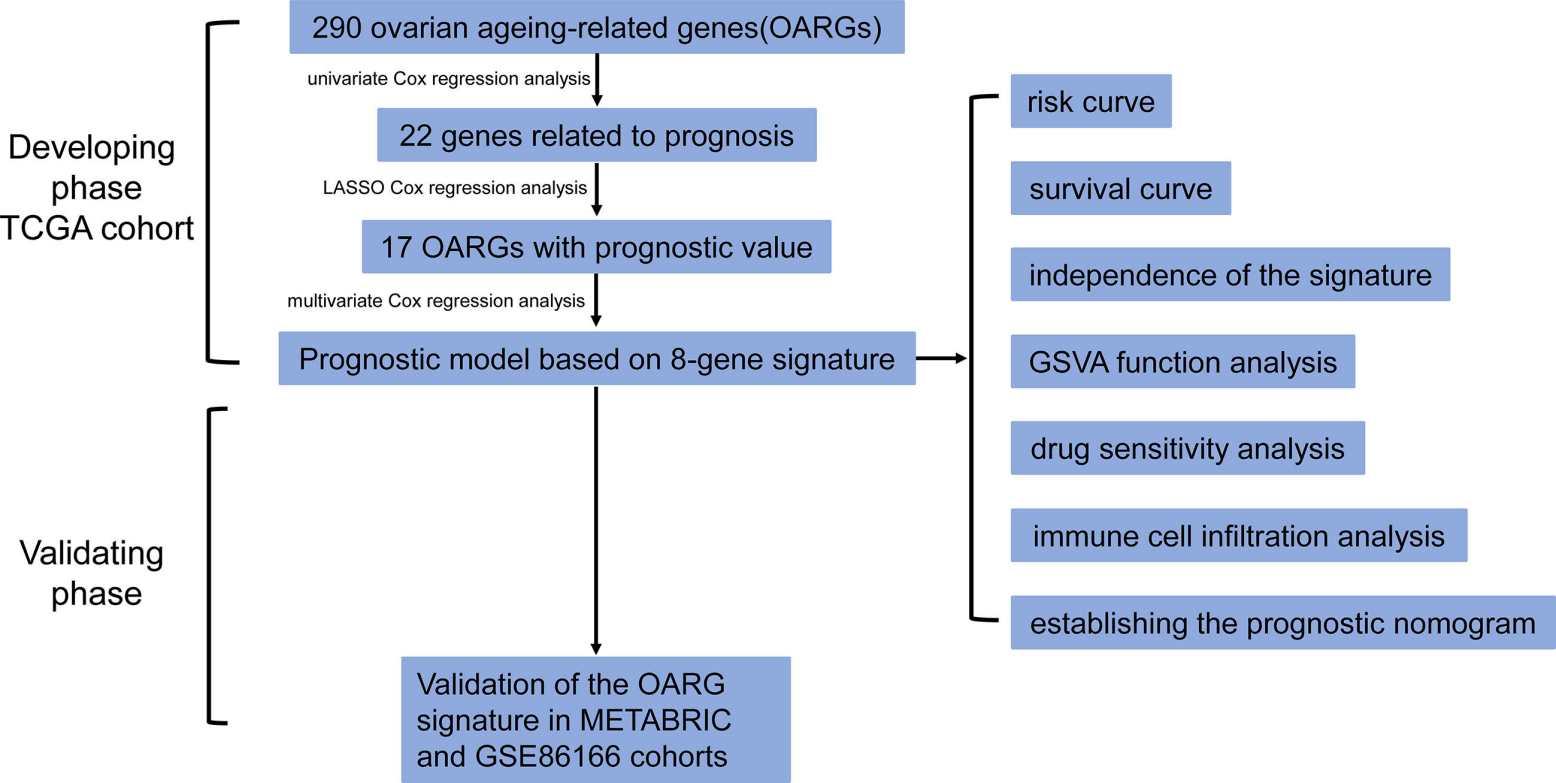
The flow chart detailing the comprehensive analysis of ovarian aging patterns in postoperative breast cancer patients.

RNA-sequencing (HTSeq-fragments per kilobase per million [FPKM]), clinicopathological, and survival data were obtained from three individual large breast cancer cohorts, namely The Cancer Genome Atlas (TCGA) database (https://portal.gdc.cancer.gov/repository, accessed in July 2022), The Molecular Taxonomy of Breast Cancer International Consortium (METABRIC) (https://www.cbioportal.org/, accessed in July 2022) and the GSE86166 dataset from Gene Expression Omnibus database (https://www.ncbi.nlm.nih.gov/geo/, accessed in July 2022). Subjects who met the following criteria were included in the study: (a) had a histologically confirmed breast cancer without metastatic disease; (b) from post-surgery; (c) with available follow-up data of overall survival (OS), and an OS of not less than 30 days. The OS was defined as the time from the date of diagnosis to the date of death due to any cause or to the date of the last follow-up. A total of 290 OARGs were identified from the study of Ruth et al. (**Table S1**) (12). The overall workflow followed in this study was presented in **Figure 1**.

### Screening for prognostic genes

The Kaplan-Meier and univariate Cox regression analyses, using OS as an outcome, were employed to estimate the predictive values of the 290 OARGs and screen for prognostic genes (with both *P* < 0.05) in the TCGA cohort.

### The prognostic pattern of ovarian aging in breast cancer

Consensus cluster analysis was carried out based on the identified prognostic genes to classify patients into different groups by a non-negative matrix factorization (NMF) algorithm using the NMF package (13). This was done to ensure maximum differences between the groups and minimum differences within the groups. The samples were clustered using the Brunet criterion. The K’s range was set at 2 to 10. According to cophenetic, dispersion, and silhouette, the ideal K was found. The prognostic pattern of ovarian aging in breast cancer

### Development and validation of the prognostic OARG signature

To further screen candidate genes for the prognostic model, the identified prognostic genes were subjected to LASSO Cox regression analysis to avoid potential co-linearity and simplify the number of independent variables (14). Then, multivariate Cox regression analysis was performed to evaluate the prognostic contributions of the selected candidate genes from the LASSO Cox regression analysis (hazard ratio, HR, 95% confidence interval, CI should not cross HR 1; *P* < 0.05), and establish the OARG risk score using the following formula: risk score = sum (each OARG normalized expression level × corresponding coefficients). Based on this, we calculated the OARG risk score for each patient and determined the optimal cut-off value for the OARG risk score according to maximally selected rank statistics method with OS for an outcome (15). Thus, according to the cutoff value, we divided each patient into different risk-stratified groups: the patient would be assigned into high-risk group if the patient’s calculated OARG risk score was larger than the cutoff value; otherwise assigned into low-risk group. The survival differences between the two risk groups were compared using Kaplan-Meier analyses with a log-rank test. Furthermore, in the TCGA cohort, a nomogram was constructed, which incorporated the OARG risk score and additional prognostic clinicopathological characteristics identified from the multivariate Cox regression analysis. Calibration curves for the survival probability at one, three, and five years were also plotted to assess the prognostic precision of this nomogram. The same procedures and calculations were performed in the METABRIC and GSE86166 cohorts for validation.

### Functional enrichment analysis of the OARG signature

Gene Set Variation Analysis (GSVA) using the “GSVA” package and Gene Set Enrichment Analysis (GSEA, https://www.gsea-msigdb.org/gsea/index.jsp) were conducted to determine the pathway and biological function differences between the two risk groups (16, 17). We used the c2.cp.kegg.v7.4.symbols.gmt in the Molecular Signatures Database (MSigDB) for board hallmarkers (17). Gene sets with normal *P* < 0.05 and false discovery rate < 0.10 were considered to be significantly enriched. Gene ontology (GO) enrichment analysis was performed using Metascape (https://metascape.org/gp/index.html#/main/step1) and plotted using the “ClusterProfiler” and “Cytoscape” package.

### Identification of potential target drugs for high-risk group patients

The “pRRophetic” package, which was developed upon statistical models calculated from huge collections of cancer cell lines gene expression and drug sensitivity data (18), was used to predict the drug sensitivity of the two risk groups. The half maximal inhibitory concentrations (IC50) of potential target drugs were compared between the two risk groups.

### Estimation of the immune cell infiltration landscape

The “GSVA” package with single-sample GSEA (ssGSEA) was used to evaluate the infiltration scores of immune cell types and immune-related pathways between the two risk groups. In addition, the variations in the compositions of immune cell types between the two risk groups were evaluated using the CIBERSORT method (19). Then, the differences in the reported famous six immune subtypes of wound healing (Immune C1), IFN-γ dominant (Immune C2), inflammatory (Immune C3), lymphocyte depleted (Immune C4), immunologically quiet (Immune C5), and TGF-β dominant (Immune C6) subtypes (20) were compared between the two groups. We also estimated the immunogenicity and immunome infiltration characteristics of breast cancer using the Estimation of STromal and Immune cells in MAlignant Tumours using Expression data (ESTIMATE) and Tumor Immune Dysfunction and Exclusion (TIDE) approaches (21, 22), and further investigated how well the risk signature performed in predicting the effects of immunotherapy. More specifically, a higher TIDE score means a higher likelihood of immune escape and a lower likelihood that the patient will benefit from immunotherapy.

### Statistical analysis

Continuous data were reported as medians with interquartile ranges (IQR), while categorical data were reported as frequencies with percentages, and compared using the Mann-Whitney U test, chi-square test, continuity corrected chi-square test, or Fisher’s exact test, whichever is appropriate. Disease-free survival (DFS) was defined as the time from the date of diagnosis to the date of recurrence/metastasis or to the date of death due to any cause or to the last follow-up. Meanwhile, recurrence-free survival (RFS) was defined as the time from the date of diagnosis to the date of recurrence or to the date of death due to any cause or to the last follow-up. The survival outcomes were estimated using the Kaplan-Meier method and compared by the log-rank test. The Cox proportional hazards model was performed to calculate the adjusted HRs and corresponding 95% confidence intervals (CIs). All statistical analyses were conducted with R version 4.1.2 (http://www.r-project.org). Statistical significance was set at two‐sided *P* < 0.05.

## Results

### Screening for prognostic OARGs

A total of 1096 subjects from the TCGA cohort, 1904 subjects from the METABRIC cohort, and 366 subjects from the GSE86166 cohort were included in this study. After filtering out subjects who did not meet our selection criteria, a total of 3267 subjects were enrolled in the final analysis, including 1017 subjects in the TCGA cohort for training, as well as1888 subjects in the METABRIC cohort and 362 subjects in the GSE86166 cohort for validation.

The Kaplan-Meier and univariate Cox regression analyses, using OS as an outcome, were conducted to screen for prognostic genes among the 290 OARGs. In total, the expression of 22 genes was found to be significantly related to OS, with 11 genes having a negative association and 11 genes with a positive association (**Figure S1**).

### The prognostic pattern of ovarian aging in breast cancer

The selected 22 prognostic OARGs were subjected to cluster analyses using the Brunet selection criterion for 50 iterations. The classification of clusters (K) was limited to 2-10. Three were chosen as the optimal cluster number based on the homogeneity, discreteness, and silhouette (**Figures S2A, B**). The results show that the OS (*P* < 0.001; **Figure S2C**) and DFS (P < 0.001; **Figure S2E**) of C2 were worse than those of C1 and C3.

### Development and validation of the prognostic OARG signature

**Figure 2:**
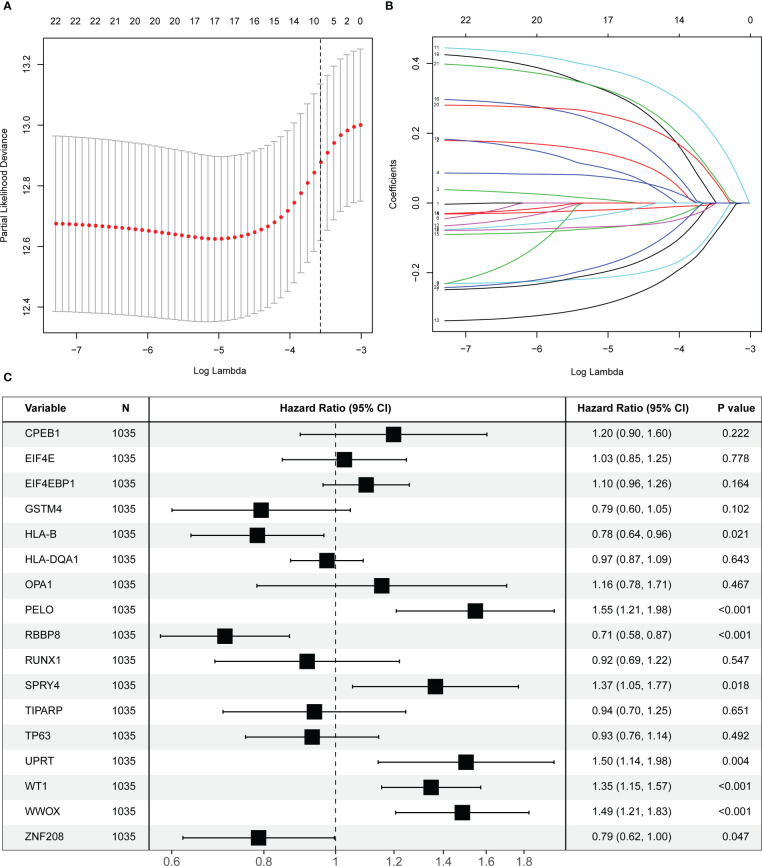
Screening and identification of prognostic ovarian ageing-related genes (OARGs) in the TCGA cohort. **(A)** Selection of the optimal candidate genes in the LASSO model. **(B)** LASSO coefficients of prognosis-associated OARGs, each curve represents a gene. **(C)** Forest plots showing results of univariate Cox regression analysis between the candidate OARGs expression and overall survival.

**Figure 3:**
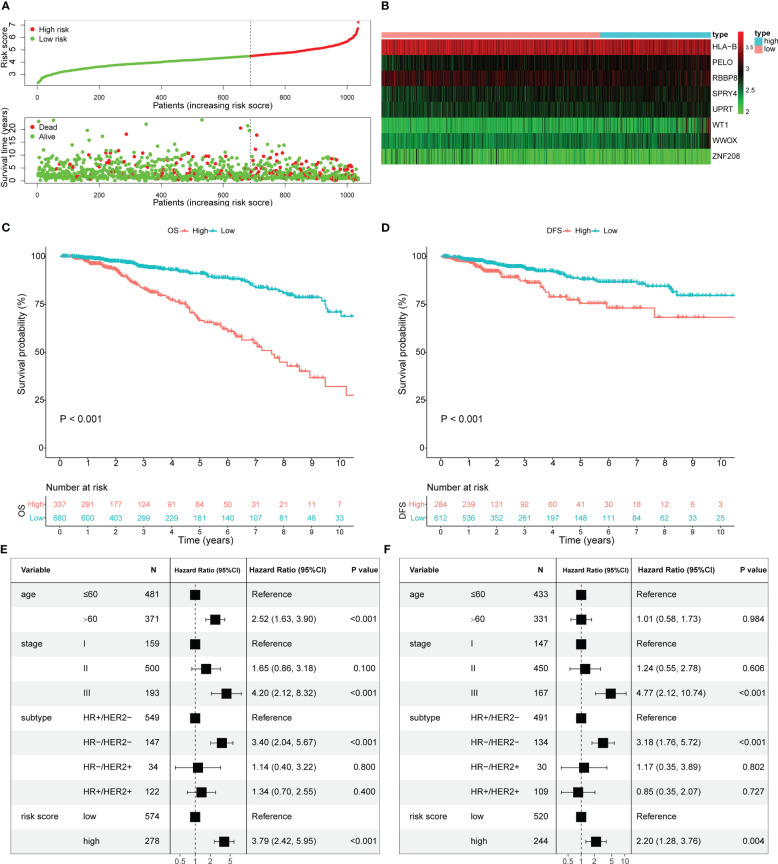
Estimate the prognostic value of ovarian ageing-related gene (OARG) signature model in TCGA cohort. **(A)** The distribution of risk scores in the TCGA and patient distribution in the high- and low-risk group according to overall survival (OS) status. **(B)** The heatmap showing expression profiles of the 8 OARGs. **(C)** Kaplan-Meier curves for the OS of patients in the high- and low-risk groups. **(D)** Kaplan-Meier curves for the diseases-free survival (DFS) of patients in the high- and low-risk groups. **(E)** Multivariate Cox regression analysis of OS. **(F)** Multivariate Cox regression analysis of DFS.

The selected 22 prognostic OARGs were also subjected to LASSO Cox regression analysis to avoid potential co-linearity and simplify the number of independent variables in the prognostic signature (**Figures 2A, B**). Subsequently, the LASSO Cox analysis yielded a total of 17 genes and therefore multivariate Cox regression analysis was performed to establish the prognostic OARG signature (**Figure 2C**). Finally, an 8-OARG risk signature was established in the TCGA cohort. The corresponding risk score of each patient was calculated using the following formula: risk score = *HLA-B* × (-0.24351) + *RBBP8* × (-0.34470) + *SPRY4* × 0.31174 + *WT1* × 0.29836 + *WWOX* × 0.39556 + *UPRT* × 0.40719+ *PELO* × 0.43603+ *ZNF208* × (-0.23972). The patients in the TCGA cohort were grouped into risk-stratified groups (high-risk group, n = 337; low-risk group, n = 680) based on the cut-off value of 4.49 which was determined using maximally selected rank statistics (**Figure S2**). The distributions of patient risk score and survival status, as well as each patient’s 8-OARGs expression levels, are summarized in **Figures 3A, B**, respectively. The Kaplan-Meier survival curves demonstrated that the high-risk group patients had significantly worse survival OS (*P <* 0.001; **Figure 3C**) and DFS (*P <* 0.001; **Figure 3D**) than the low-risk group patients. Moreover, the OARG risk signature remained significantly associated with OS (HR = 3.79, 95% CI = 2.42-5.95, *P <* 0.001; **Figure 3E**) and DFS (HR = 2.20, 95% CI = 1.28-3.76, *P* = 0.004; **Figure 3F**) after adjusting for other clinicopathological variables.

Using the same formula and the cut-off value from the TCGA cohort, the risk scores and risk-stratified groupings weredetermined for patients in the METABRIC and GSE86166 cohorts for validation (**Figures S3**, **S4**). Consistently, the Kaplan-Meier survival curves also showed that the high-risk group patients had significantly worse OS (*P <* 0.001; **Figure S3C**) and RFS (*P <* 0.001; **Figure S3D**) in the METABRIC cohort, and worse OS (*P =* 0.016; **Figure S4C**) and RFS (*P =* 0.022; **Figure S4D**) in the GSE86166 cohort, respectively. Furthermore, after adjusting for other clinicopathological variables, the OARG risk signature remained associated with OS (HR = 1.35, 95% CI = 1.14-1.60, *P <* 0.001; **Figure S3E**) and RFS (HR = 1.22, 95% CI = 1.00-1.49, P = 0.050; **Figure S3F**) in the METABRIC cohort and OS (HR = 1.94, 95% CI = 1.05-3.60, P = 0.035; **Figure S4E**) and RFS (HR = 1.86, 95% CI = 0.91-3.82, P = 0.090; **Figure S4F**) in the GSE86166 cohort, respectively.

### Establishment of a prognostic nomogram based on the OARG signature

**Figure 4:**
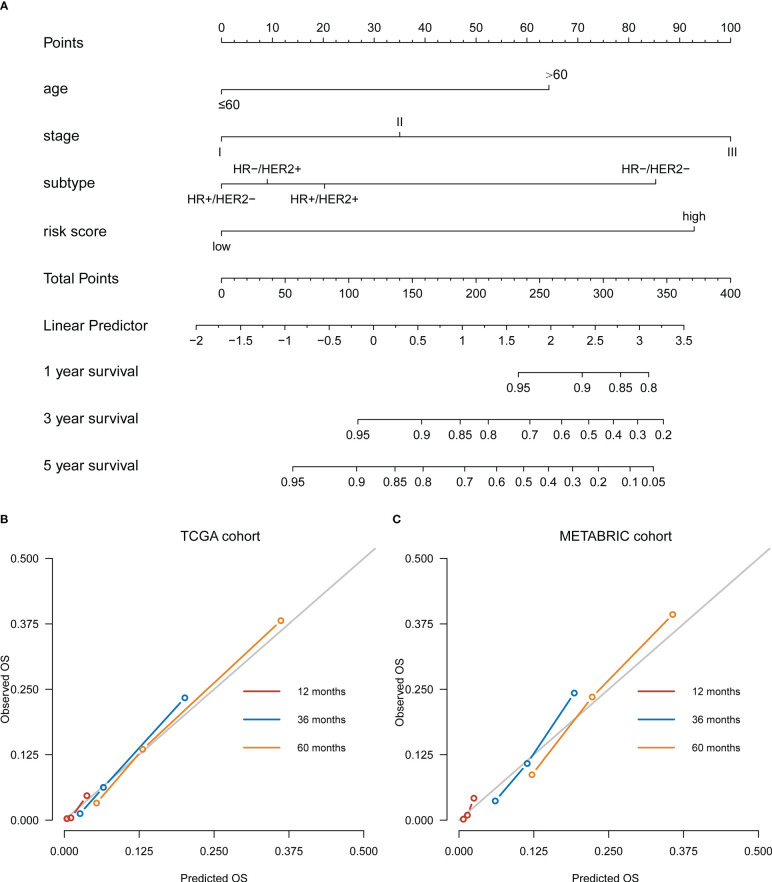
Development of a nomogram based on ovarian ageing-related genes (OARGs) signature for predicting overall survival (OS) of patients with breast cancer. **(A)** The nomogram plot integrating OARG risk score, age, stage and subtype in the TCGA training cohort. **(B)** The calibration plot for the probability of 1-, 3-, and 5-year OS in the TCGA training cohort. **(C)** The calibration plot for the probability of 1-, 3-, and 5-year OS in the METABRIC validation cohort.

A risk score-based visualized nomogram, which integrates the risk signature and three important clinicopathological factors (age, stage and subtype) selected from the multivariate Cox regression analysis, was established to individually quantify and assess the OS probability at 1-, 3- and 5-years of breast cancer patients in TCGA cohort (**Figure 4A**). We conducted a bootstrap validation and calculated the nomogram’s C-index to be 0.812 (95% CI: 0.768-0.856) in the TCGA cohort and 0.757 (95% CI: 0.734-0.779) in the METABRIC cohort, respectively. To evaluate the predictive efficacy and clinical application of the nomogram, calibration curves were plotted for both the TCGA cohort (**Figure 4B**) and the METABRIC cohort (**Figure 4C**). The calibration curves demonstrated satisfactory consistency among the actual and anticipated OS probabilities at 1-, 3- and 5-years.

### Gene set variation analysis of OARG signature

We performed GSVA to determine the potential biological functions of the OARG signature in breast cancer. In the training cohort of TCGA, the pathway sets DNA sensing, primary immunodeficiency, and nutrients metabolism were found to be activated in the high-risk group (**Figure S5A**). Meanwhile, the pathway sets with the immune network, autoimmune system, and immune disease were activated in the low-risk group (**Figure S6D**). GO enrichment analysis confirmed that the immune-related biological processes were enriched in the low-risk group (**Figure S6A**). These results were further validated in the METABRIC (**Figures S5B**, **S6B, E**) and GSE86166 (**Figures S5C**, **S6C, F**) cohorts and similar functional results were found. These results support the comprehensive DNA repair and immunomodulatory function effects of the OARG signature in the development and progression of breast cancer.

### Clinical implications of the OARG signature in predicting therapeutic effects

**Figure 5:**
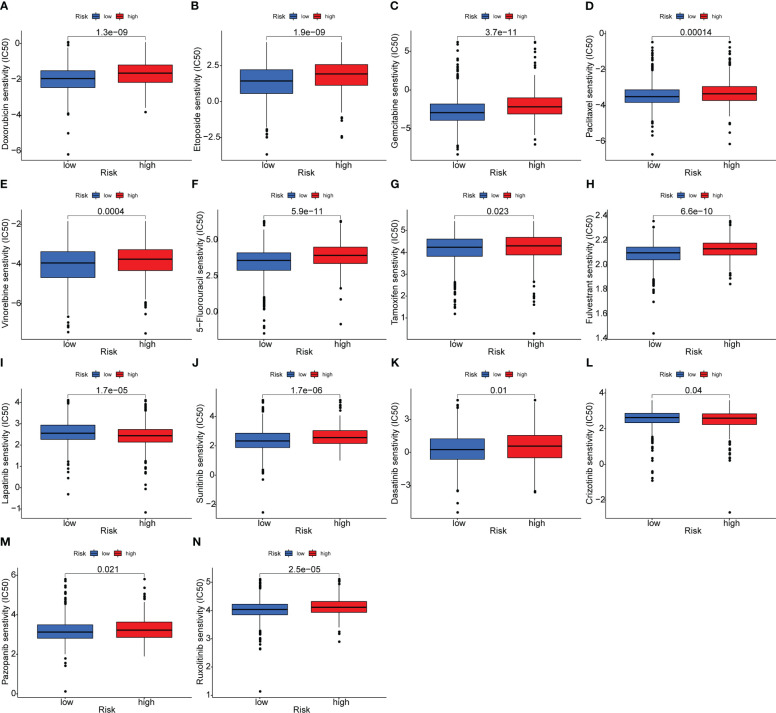
Analysis of the association between the risk model and chemotherapeutics, endocrine therapy, and targeted therapy. **(A–F)** The model predicting the sensitivity to chemosensitivity. It was estimated that low-risk patients had lower IC50 for chemotherapeutics of doxorubicin, etoposide, gemcitabine, paclitaxel, vinorelbine and 5-fluorouracil. **(G, H)** The model predicting the sensitivity to endocrine therapy. It was estimated that low-risk patients had lower IC50 of tamoxifen and fulvestrant. **(I–N)** The model predicting the sensitivity to targeted therapy. It was estimated that low-risk patients had lower IC50 of lapatinib, sunitinib, dasatinib, crizotinib, pazopanib and ruxolitinib.

The potential intrinsic connections between the OARG signature and therapeutic effects of chemotherapeutic, endocrine, and targeted agents were further explored. In the training cohort of TCGA, the low-risk group had a lower IC50 for chemotherapeutics such as doxorubicin, etoposide, gemcitabine, paclitaxel, vinorelbine and 5-fluorouracil, indicating the predictive potential of the model for chemosensitivity (**Figures 5A–F**). For the endocrine and targeted drugs, the low-risk patients had a lower IC50 for tamoxifen and fulvestrant (**Figures 5G, H**), as well as for lapatinib, sunitinib, dasatinib, crizotinib, pazopanib, and ruxolitinib (**Figures 5I–N**). Most of the results were validated in the METABRIC (except for crizotinib; **Figure S7**) and the GSE86166 (except for vinorelbine, crizotinib, and ruxolitinib; **Figure S8**) cohorts. The better prognosis for the low-risk group could be partially explained by these findings. These findings also imply that the low-risk group would benefit more from therapy with traditional and novel drugs.

### Immunocyte infiltration profiling of the OARG signature in breast cancer

**Figure 6:**
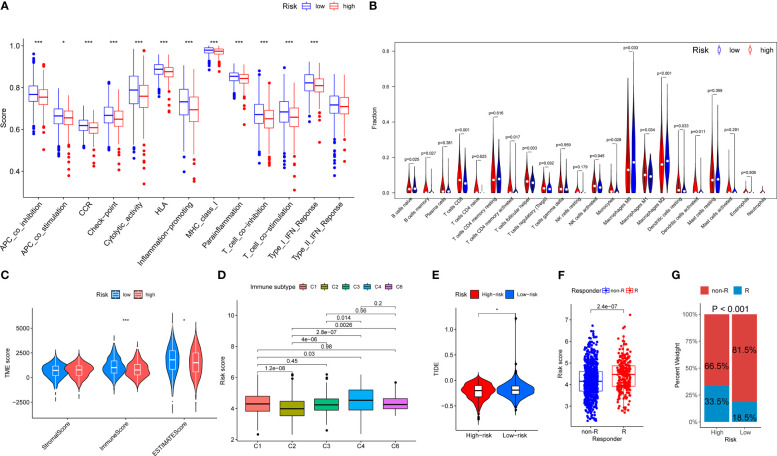
The landscape of immune function and immune cell infiltration between the high- and low-risk group in the TCGA cohort. Red represents high-risk samples; blue represents low-risk samples. *P < 0.05, **P < 0.01, ***P < 0.001. **(A)** Barplot showing differences of immune functions between the low- and the high-risk group. **(B)** Violin plot showing differences of infiltrating immune cell types between the low- and the high-risk group. **(C)** Comparison of tumor microenvironment scores calculated by ESTIMATE between the low- and the high-risk group. **(D)** Comparison of risk scores between different immune subgroups. **(E)** Comparison of tumor microenvironment scores calculated by TIDE between the low- and the high-risk group. **(F)** Comparison of risk scores between different responder subgroups. **(G)** Comparison of the immunotherapy responding proportion between the low- and the high-risk group.

The profiling of immune infiltration was performed using the ssGSEA and CIBERSORT methods, and the outcomes showed noticeably different immune infiltration landscapes between the two risk categories. Specifically, functions such as APC_co_inhibition, APC_co_stimulation, CCR, Check-point, Cytolytic_activity, HLA, Inflammation-promoting, MHC_class_I, Parainflammation, T_cell_co-inhibition, T_cell_co-stimulation and Type_I_IFN_Reponse were elevated in the low-risk group patients (**Figure 6A**). Moreover, the patients in the low-risk group exhibited a higher percentage of B cells naive, Macrophages M0 and Macrophages M2. In contrast, the percentages of B cells memory, T cells CD8, T cells CD4 memory activated, T cells follicular helper, NK cells activated, Monocytes, Macrophages M1, Dendritic cells resting and Dendritic cells activated were all higher in high-risk group individuals (**Figure 6B**). In addition, the high-risk group had significantly lower immune and ESTIMATE scores than the low-risk group (**Figure 6C**). There was no immune C5 subtype in our cohort and the risk scores between the immune subtypes significantly differed. The immune C4 subtype had the highest risk score and the immune C2 subtype had the lowest risk score (**Figure 6D**). In contrast, the low-risk group presented with higher TIDE scores indicating that the low-risk group patients may be more susceptible to immune escape (**Figure 6E**). The patients responding to immunotherapy also had higher risk scores than those non-responding to immunotherapy (**Figure 6F**). We also discovered that the proportion of patients responding to immunotherapy in the high-risk group was higher than that in the low-risk group (33.5% vs 18.5%, *P* < 0.001, **Figure 6G**). Overall, these findings showed that the immune infiltration profiles in breast cancer are linked with the risk stratification based on the OARG signature, and the immunotherapy effects could be also predicted.

## Discussion

The current multicohort genetic association research provided a bioinformatics-based analysis model, which incorporated clinical information collection, transcriptome profiling, survival analysis, functional evaluation, and immune infiltration estimation to interpret the possible molecular mechanisms of ovarian aging and its implication in breast cancer. Moreover, this analysis model proposes a comprehensive perspective to explore the ovarian aging microenvironment in breast cancer and could reveal the potential outcomes and mechanisms related to the prognostic OARG signature.

Ovarian aging, involves complex genetic variants regulation and elaborate biological mechanisms. It is linked to several unfavorable consequences of hormone-sensitive cancers (23, 24). In recent years, increasing evidence suggests that ovarian aging is crucial in the female reproductive longevity biological processes, which have been demonstrated to be associated with the tumorigenesis and development of endocrine tumors (25–29). This study developed a signature featuring 8 OARGs (HLA-B, RBBP8, SPRY4, WT1, WWOX, UPRT, PELO, ZNF208) and determined its prognostic and functional implications in breast cancer patients. HLA-B has been previously demonstrated to have significant immunogenic involvement in breast cancer by supporting multiple downstream immunogenic pathways (30, 31). Our research showed that a better prognosis was related to a relatively higher expression of HLA-B. On the other hand, RBBP8 functions as a tumor suppressor protein in breast cancer by interacting with some distinct tumor-suppressing factors, including BRCA1 and retinoblastoma (32, 33). Our findings also suggest that RBBP8 served as a protective factor for breast cancer. An *in vivo* research revealed that SPRY4 may influence the characteristics of cancer stem cells, as well as tumor cell migration and proliferation (34). Numerous studies have demonstrated that WT1 plays an oncogenic role in various solid cancers including breast cancer, by promoting epithelial-to-mesenchymal transition and lowering chemotherapy efficacy (35, 36). Although previous studies found that WWOX expression was reduced in various cancers, our study has shown that it may be a risk factor affecting the prognosis of breast cancer (37). Moreover, the current study found that the overexpression of UPRT was associated with a worse prognosis in breast cancer and is closely related to cancer gene-therapy efficacy (38). PELO is a new HER-signaling regulator and was suggested to play a role in inhibiting tumor cell proliferation and metastasis (39, 40). ZNF208 is a member of the zinc finger family of proteins and its mutations were found in many cancers, such as pancreatic cancer, gastric cancer, esophageal cancer and laryngeal cancer (41–43). We discovered its prognostic significance for breast cancer in our investigation.

The functional analysis results support the comprehensive DNA damage repair and immunomodulatory functions of the OARG signature in the development and progression of breast cancer. DNA damage repair mechanisms can trigger an innate immune response, resulting in a reduction in cell proliferation and the production of interferon, which is a crucial mechanism for promoting immune regulation (44–46). The tumor microenvironment enables tumor cells to avoid immune monitoring and medication interference, which permits them to survive (47). Previous studies have found that numerous pathways and genes associated with DNA damage repair networks play a role in genetic instability and immune activity (46, 48–50). Our results revealed that patients in the low-risk group exhibited a higher percentage of B cells naive, Macrophages M0 and Macrophages M2. Macrophages M0 have been polarized into M1-like and M2-like subtypes, both of these two macrophages are strongly linked to inflammatory reactions. Specifically, M1-like macrophages are primarily involved in pro-inflammatory reactions, while M2-like macrophages primarily participate in anti-inflammatory reactions (51). Ovarian aging activity is typically connected to the trigger of the anti-inflammatory signal, which is consistent with our results. Many studies have revealed that a better outcome is associated with the abundance of M1-like macrophages, while a worse outcome is suggested by the predominance of M2-like macrophages in breast cancer (52, 53). Therefore, the increased enrichment of M2-like macrophages that occurs with ovarian aging may be a possible explanation for the poor prognosis and may serve as a novel prognostic biomarker for breast cancer. Additionally, patients in the low-risk group had lower IC50 values for chemotherapeutic agents (doxorubicin, etoposide, gemcitabine, paclitaxel, vinorelbine, and 5-fluorouracil), endocrine agents (tamoxifen and fulvestrant), and targeted agent (lapatinib), which may have contributed to their better prognosis, since they were more responsive to systemic therapeutic drugs. Moreover, patients in the low-risk group have a higher sensitivity to sunitinib, pazopanib, ruxolitinib and crizotinib, which are currently being tested in ongoing clinical trials and may be potential targets for breast cancer therapy.

Although the present study shows that the OARG signature has an excellent performance in multicohort of breast cancer datasets, the study also has some limitations. Firstly, the participants were retrospectively enrolled, which may inevitably introduce bias to some extent. Secondly, the functional results of OARG genes from our bioinformatics analyses have not yet been confirmed in *in vitro* and *in vivo* experimental studies. Thirdly, we recognize that it is essential for well-designed clinical trials to investigate the prognostic significance of this model and its therapeutic implications in selecting novel drugs for breast cancer.

In conclusion, the current multicohort genetic association research comprehensively explored the OARGs in breast cancer based on their biological functions, linked pathways, regulatory immune infiltration, efficacy levels, and clinical implications. The survival-related OARG signature proposed in the current study has the potential to distinguish prognosis and may be clinically applied as useful biomarker and candidate targets in breast cancer.

## Data availability statement

The original contributions presented in the study are included in the article/**Supplementary Material**. Further inquiries can be directed to the corresponding author.

## Author contributions

XH did the literature search. XH designed the study. XH, Q-WZ, Y-NZ, LC, M-DW, Y-SG, and J-YC participated in the analysis and interpretation of data. XH and J-YC developed an early draft. All authors contributed to the article and approved the submitted version.

## Supplementary material

Supplementary Figure 1 Screening of ovarian ageing related prognostic genes by univariate Cox regression analysis.

Supplementary Figure 2 The prognostic pattern of ovarian aging in breast cancer and determination of the optimal cutoff value of the vitamin C index according to maximally selected rank statistics.

Supplementary Figure 3 Estimate the prognostic value of ovarian ageing-related gene (OARG) signature model in METABRIC cohort. **(A)** The distribution of risk scores in the TCGA and patient distribution in the high- and low-risk group according to overall survival (OS) status. **(B)** The heatmap showing expression profiles of the 8 OARGs. **(C)** Kaplan-Meier curves for the OS of patients in the high- and low-risk groups. **(D)** Kaplan-Meier curves for the recurrence-free survival (RFS) of patients in the high- and low-risk groups. **(E)** Multivariate Cox regression analysis of OS. **(F)** Multivariate Cox regression analysis of RFS.

Supplementary Figure 4 Estimate the prognostic value of ovarian ageing-related gene (OARG) signature model in GSE86166 cohort. **(A)** The distribution of risk scores in the TCGA and patient distribution in the high- and low-risk group according to overall survival (OS) status. **(B)** The heatmap showing expression profiles of the 8 OARGs. **(C)** Kaplan-Meier curves for the OS of patients in the high- and low-risk groups. **(D)** Kaplan-Meier curves for the recurrence-free survival (RFS) of patients in the high- and low-risk groups. **(E)** Multivariate Cox regression analysis of OS. **(F)** Multivariate Cox regression analysis of RFS.

Supplementary Figure 5 Kyoto Encyclopedia of Genes and Genomes (KEGG) functional enrichment analysis of ovarian ageing-related gene (OARG) signature. **(A)** TCGA cohort. **(B)** METABRIC cohort. **(B)** GSE86166 cohort.

Supplementary Figure 6 Gene ontology (GO) and Gene set enrichment analysis (GSEA) functional enrichment analysis functional enrichment analysis of ovarian ageing-related gene (OARG) signature. GO functional enrichment analysis for **(A)** TCGA cohort. **(B)** METABRIC cohort. **(B)** GSE86166 cohort; GSEA functional enrichment analysis for **(D)** TCGA cohort. **(E)** METABRIC cohort. **(F)** GSE86166 cohort.

Supplementary Figure 7 Analysis of the association between the risk model and chemotherapeutics, endocrine therapy, and targeted therapy in the METABRIC cohort. **(A–F)** The model predicting the sensitivity to chemosensitivity. It was estimated that low-risk patients had lower IC50 for chemotherapeutics of doxorubicin, etoposide, gemcitabine, paclitaxel, vinorelbine and 5-fluorouracil. (GH) The model predicting the sensitivity to endocrine therapy. It was estimated that low-risk patients had lower IC50 of tamoxifen and fulvestrant. **(I–M)** The model predicting the sensitivity to targeted therapy. It was estimated that low-risk patients had lower IC50 of lapatinib, sunitinib, dasatinib, pazopanib and ruxolitinib.

Supplementary Figure 8 Analysis of the association between the risk model and chemotherapeutics, endocrine therapy, and targeted therapy in the GSE86166 cohort. **(A–E)** The model predicting the sensitivity to chemosensitivity. It was estimated that low-risk patients had lower IC50 for chemotherapeutics of doxorubicin, etoposide, gemcitabine, paclitaxel and 5-fluorouracil. (FG) The model predicting the sensitivity to endocrine therapy. It was estimated that low-risk patients had lower IC50 of tamoxifen and fulvestrant. **(H–K)** The model predicting the sensitivity to targeted therapy. It was estimated that low-risk patients had lower IC50 of lapatinib, sunitinib, dasatinib and pazopanib.
