# Supplementary figures and images for: The prognostic significance of human ovarian aging-related signature in breast cancer after surgery: A multicohort study
Source: Front Immunol. 2023 Mar 7;14:1139797. doi: 10.3389/fimmu.2023.1139797 (PMC10027938; doi:10.3389/fimmu.2023.1139797)

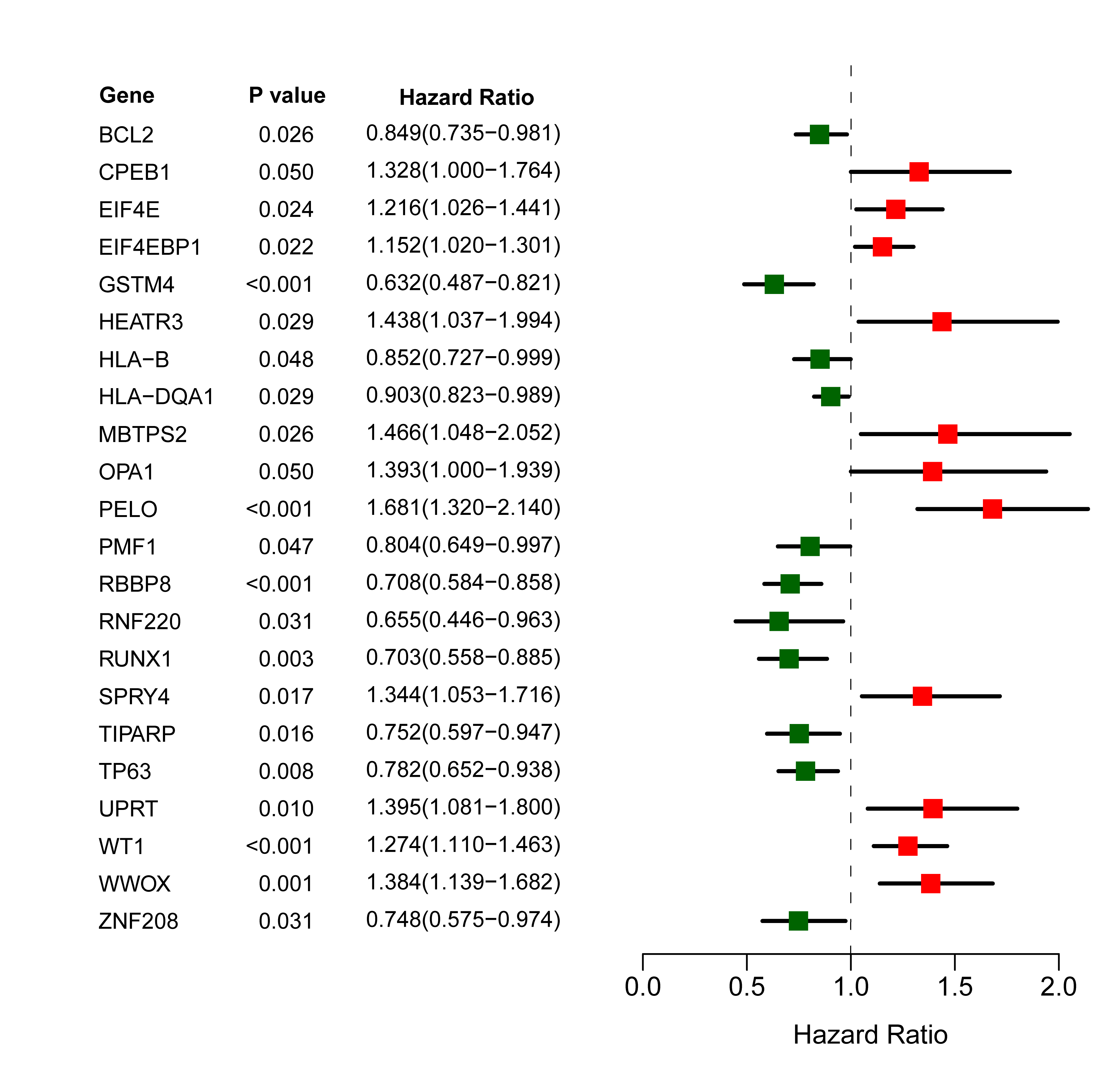

Supplement: Supplementary Figure 1 — Screening of ovarian ageing related prognostic genes by univariate Cox regression analysis. [file Image_1.tif]

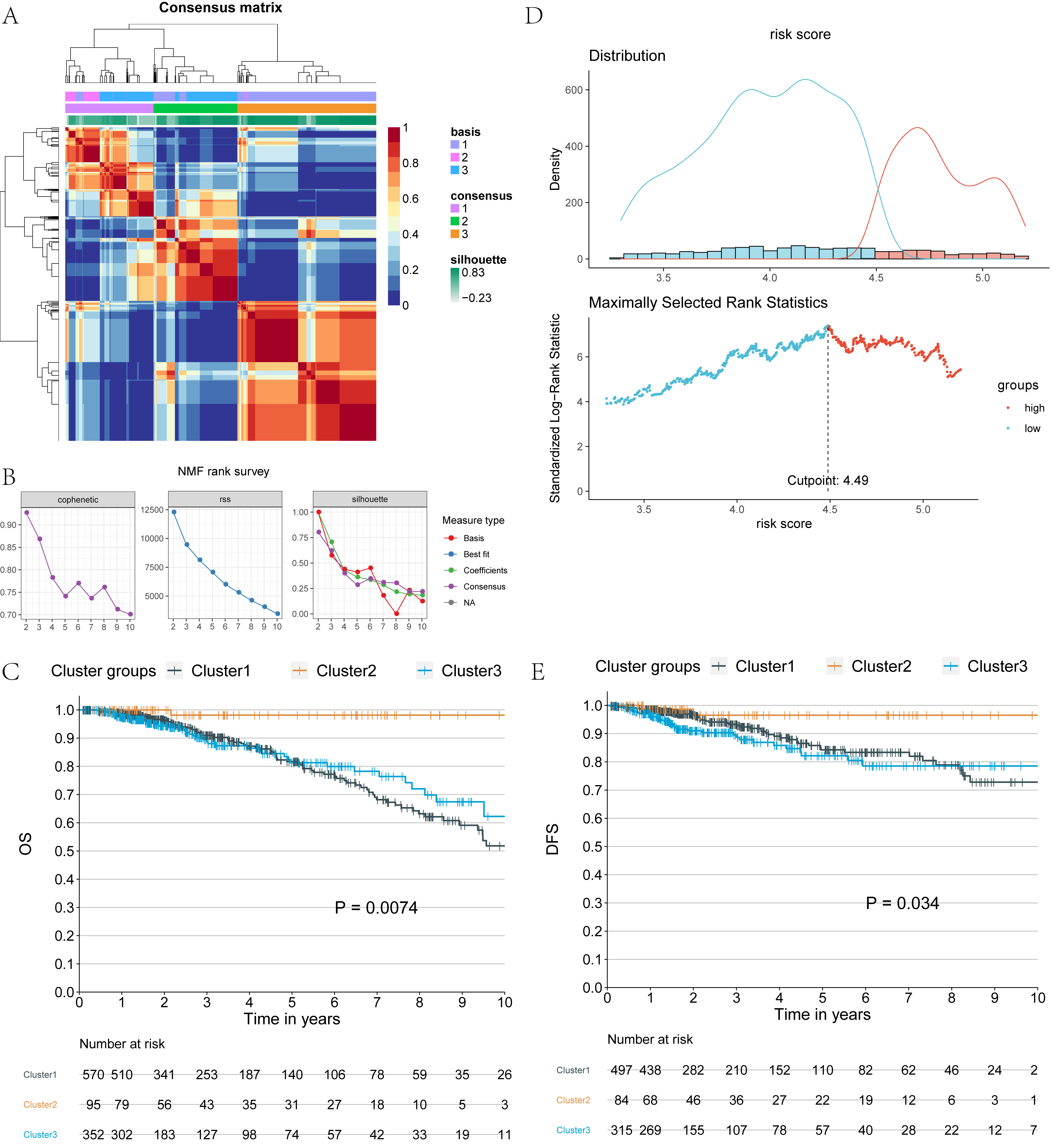

Supplement: Supplementary Figure 2 — The prognostic pattern of ovarian aging in breast cancer and determination of the optimal cutoff value of the vitamin C index according to maximally selected rank statistics. [file Image_2.tif]

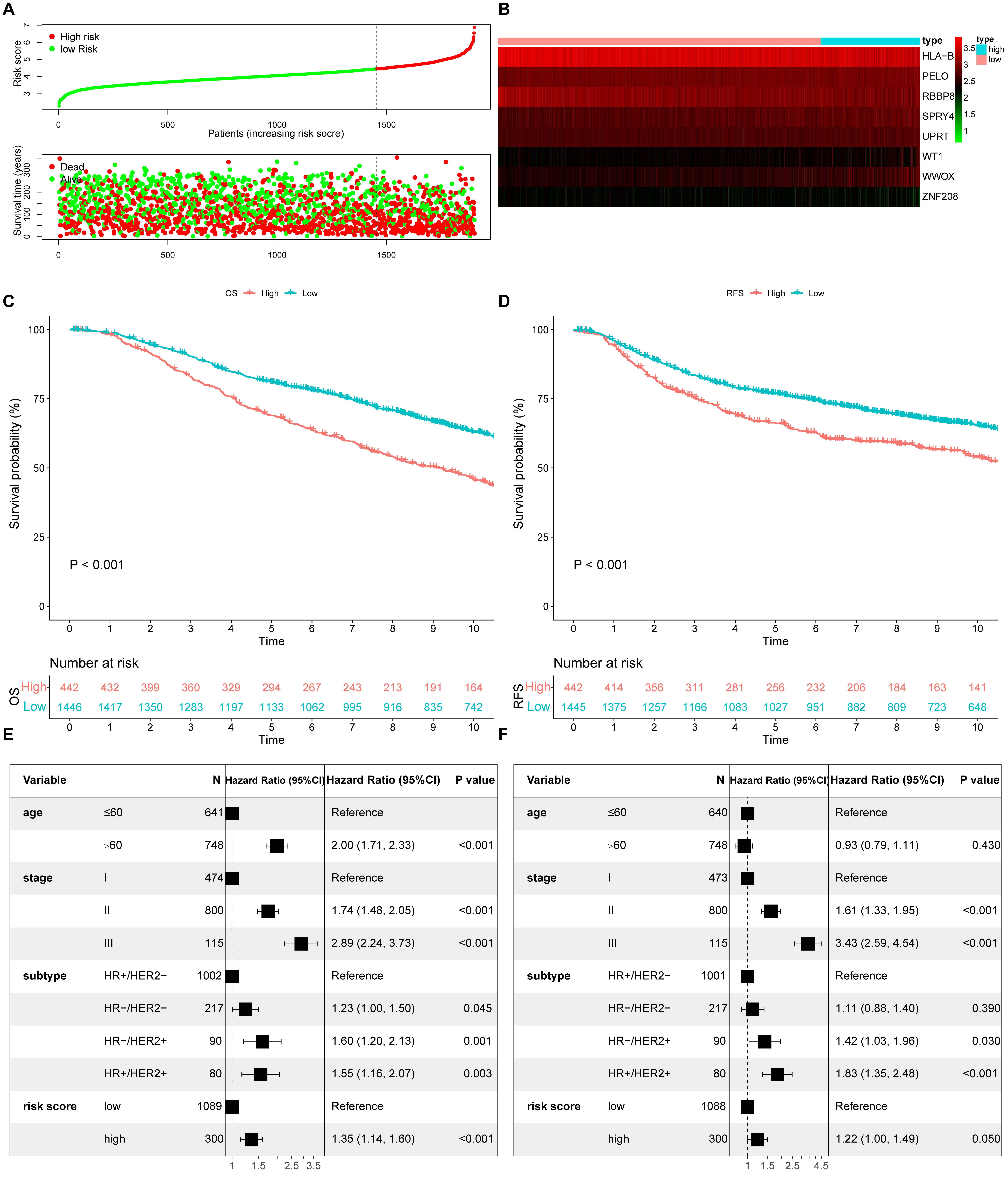

Supplement: Supplementary Figure 3 — Estimate the prognostic value of ovarian ageing-related gene (OARG) signature model in METABRIC cohort. (A) The distribution of risk scores in the TCGA and patient distribution in the high- and low-risk group according to overall survival (OS) status. (B) The heatmap showing expression profiles of the 8 OARGs. (C) Kaplan-Meier curves for the OS of patients in the high- and low-risk groups. (D) Kaplan-Meier curves for the recurrence-free survival (RFS) of patients in the high- and low-risk groups. (E) Multivariate Cox regression analysis of OS. (F) Multivariate Cox regression analysis of RFS. [file Image_3.tif]

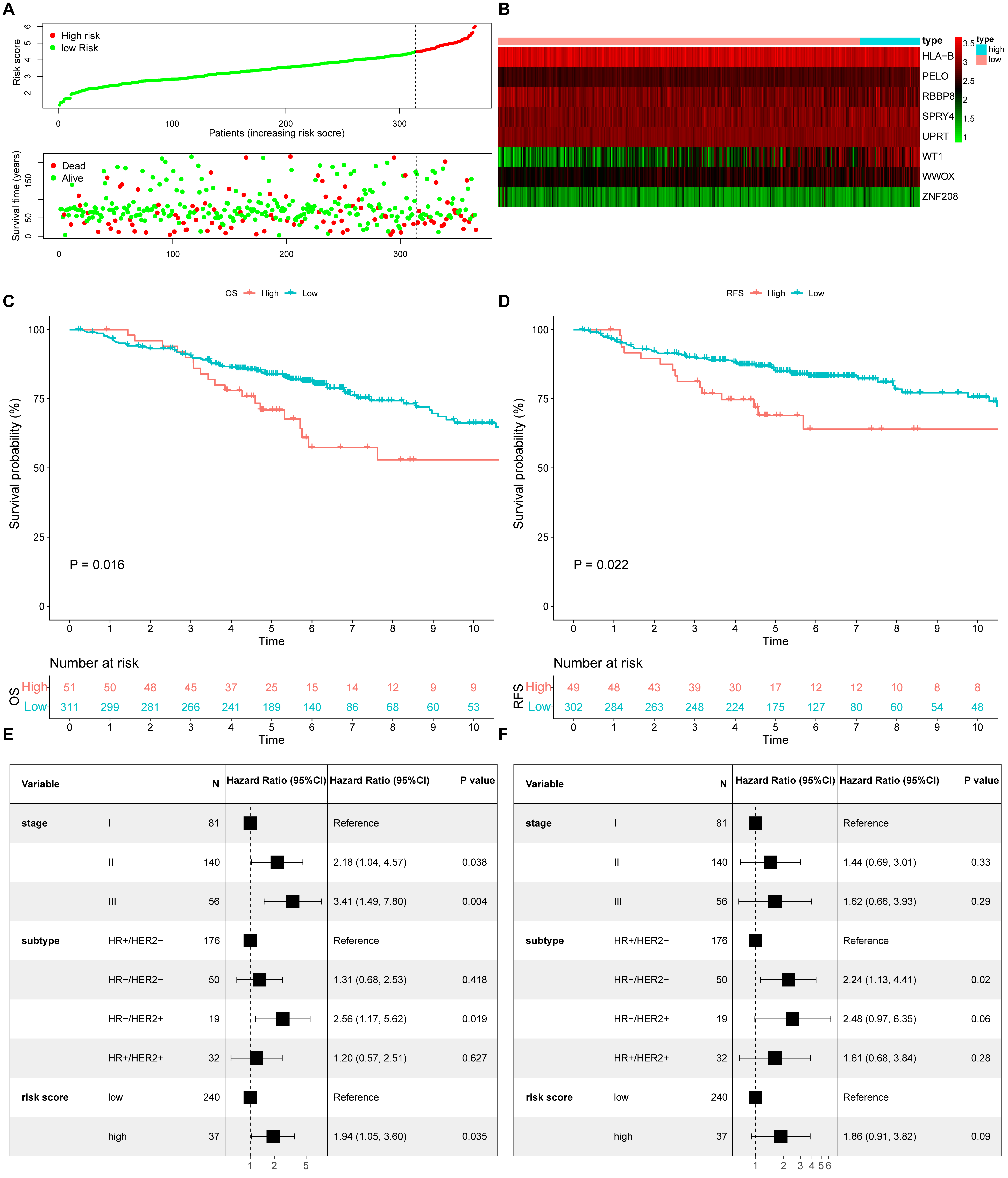

Supplement: Supplementary Figure 4 — Estimate the prognostic value of ovarian ageing-related gene (OARG) signature model in GSE86166 cohort. (A) The distribution of risk scores in the TCGA and patient distribution in the high- and low-risk group according to overall survival (OS) status. (B) The heatmap showing expression profiles of the 8 OARGs. (C) Kaplan-Meier curves for the OS of patients in the high- and low-risk groups. (D) Kaplan-Meier curves for the recurrence-free survival (RFS) of patients in the high- and low-risk groups. (E) Multivariate Cox regression analysis of OS. (F) Multivariate Cox regression analysis of RFS. [file Image_4.tif]

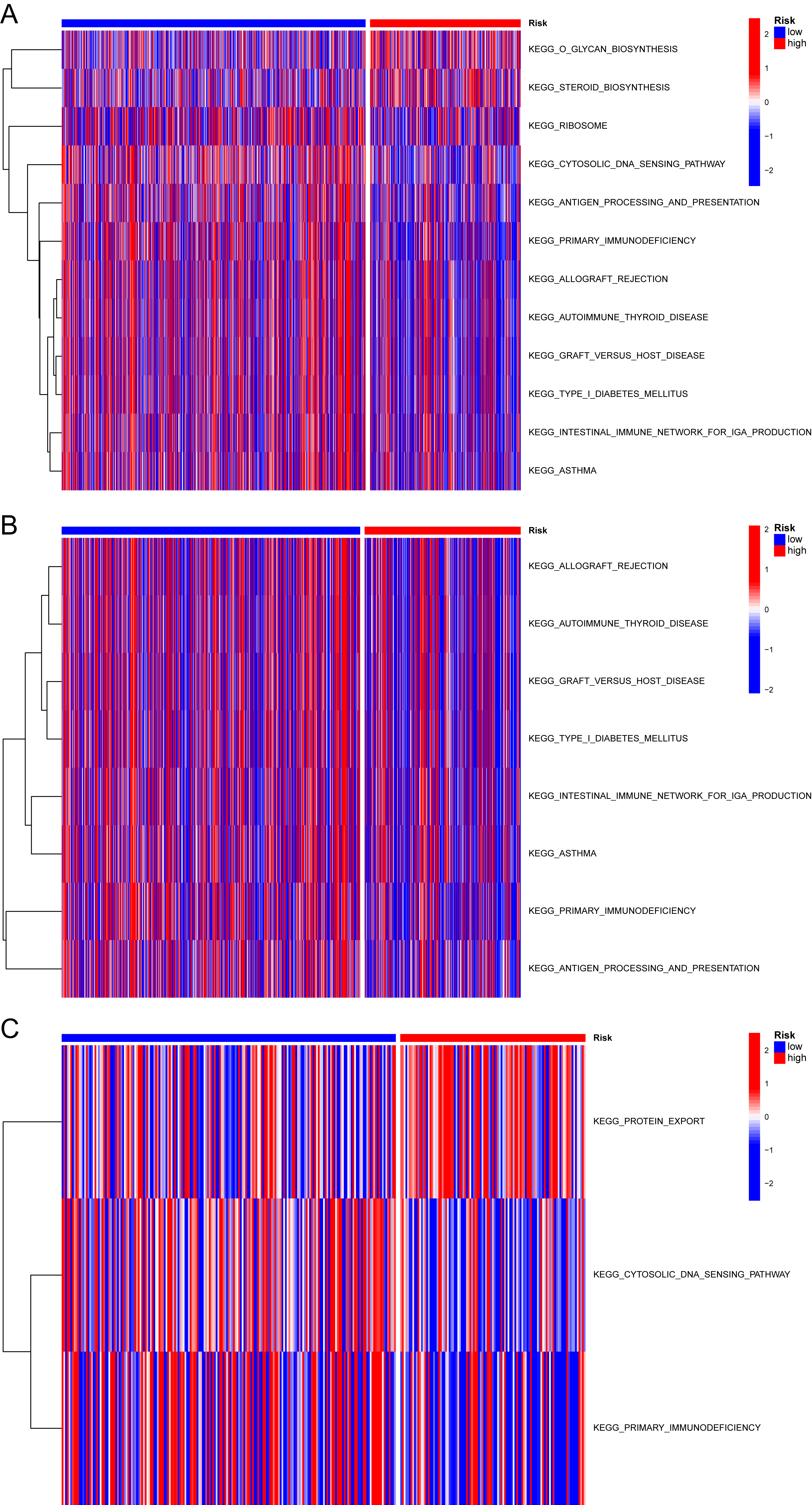

Supplement: Supplementary Figure 5 — Kyoto Encyclopedia of Genes and Genomes (KEGG) functional enrichment analysis of ovarian ageing-related gene (OARG) signature. (A) TCGA cohort. (B) METABRIC cohort. (B) GSE86166 cohort. [file Image_5.tif]

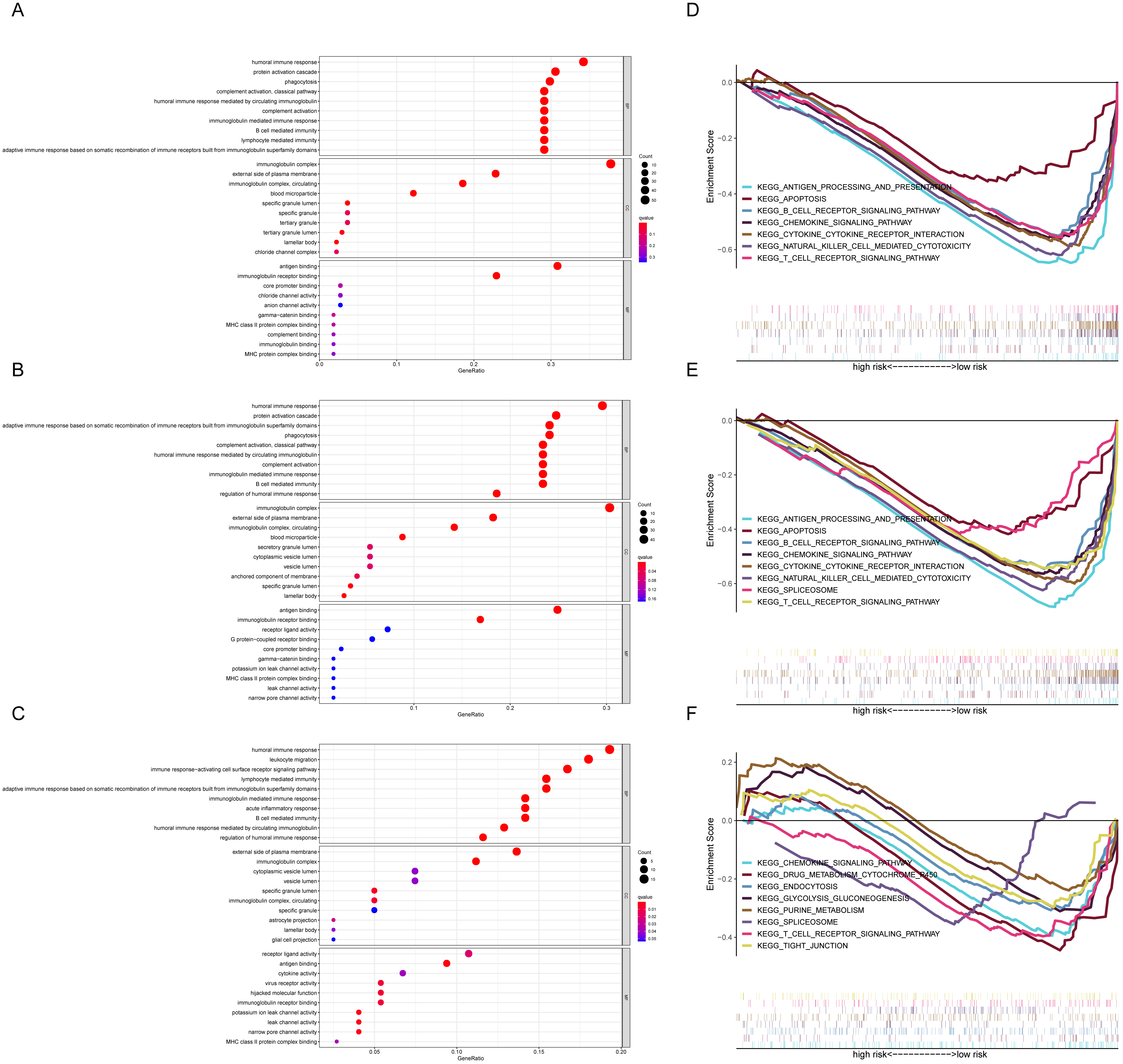

Supplement: Supplementary Figure 6 — Gene ontology (GO) and Gene set enrichment analysis (GSEA) functional enrichment analysis functional enrichment analysis of ovarian ageing-related gene (OARG) signature. GO functional enrichment analysis for (A) TCGA cohort. (B) METABRIC cohort. (B) GSE86166 cohort; GSEA functional enrichment analysis for (D) TCGA cohort. (E) METABRIC cohort. (F) GSE86166 cohort. [file Image_6.tif]

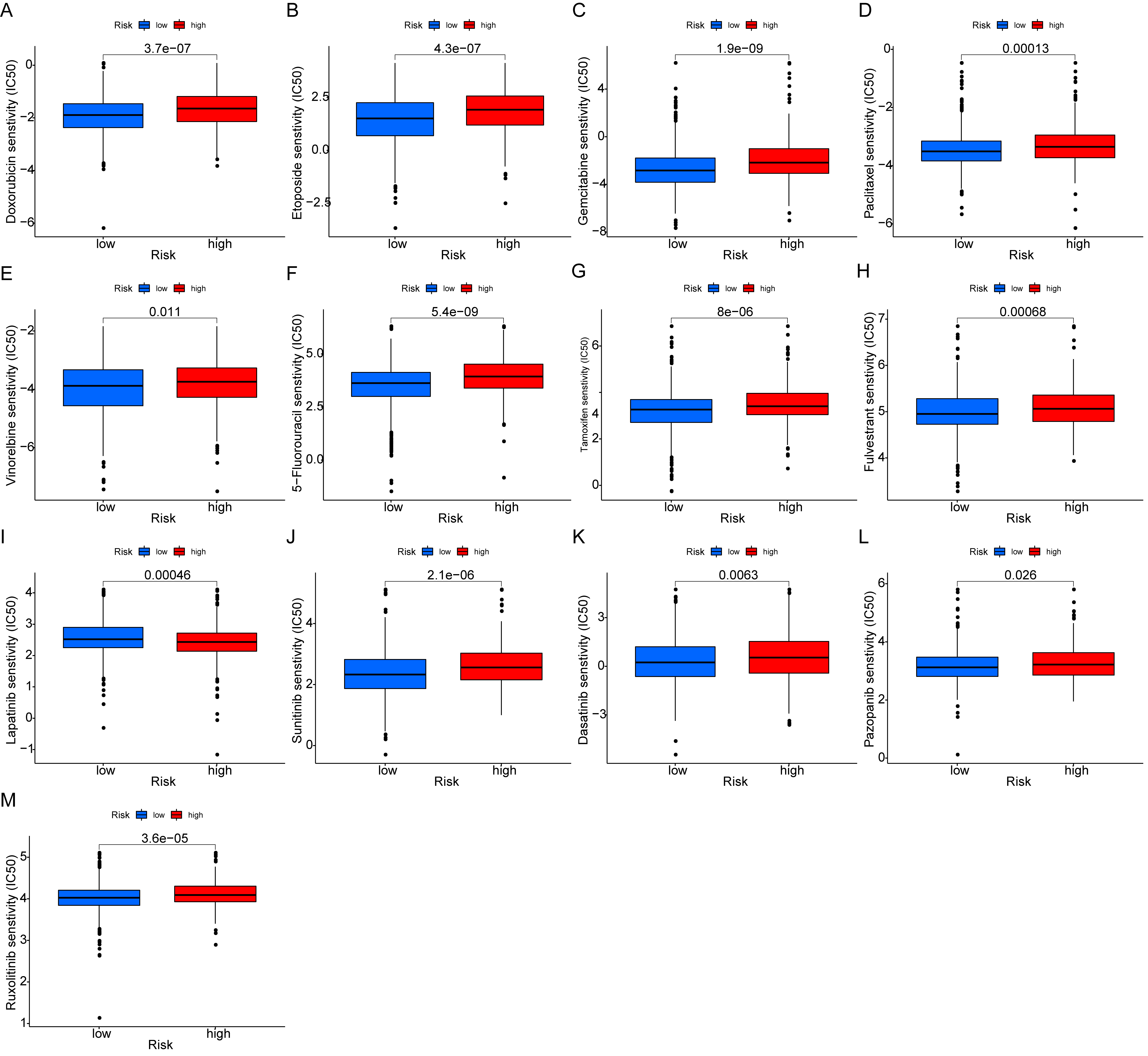

Supplement: Supplementary Figure 7 — Analysis of the association between the risk model and chemotherapeutics, endocrine therapy, and targeted therapy in the METABRIC cohort. (A–F) The model predicting the sensitivity to chemosensitivity. It was estimated that low-risk patients had lower IC50 for chemotherapeutics of doxorubicin, etoposide, gemcitabine, paclitaxel, vinorelbine and 5-fluorouracil. (GH) The model predicting the sensitivity to endocrine therapy. It was estimated that low-risk patients had lower IC50 of tamoxifen and fulvestrant. (I–M) The model predicting the sensitivity to targeted therapy. It was estimated that low-risk patients had lower IC50 of lapatinib, sunitinib, dasatinib, pazopanib and ruxolitinib. [file Image_7.tif]

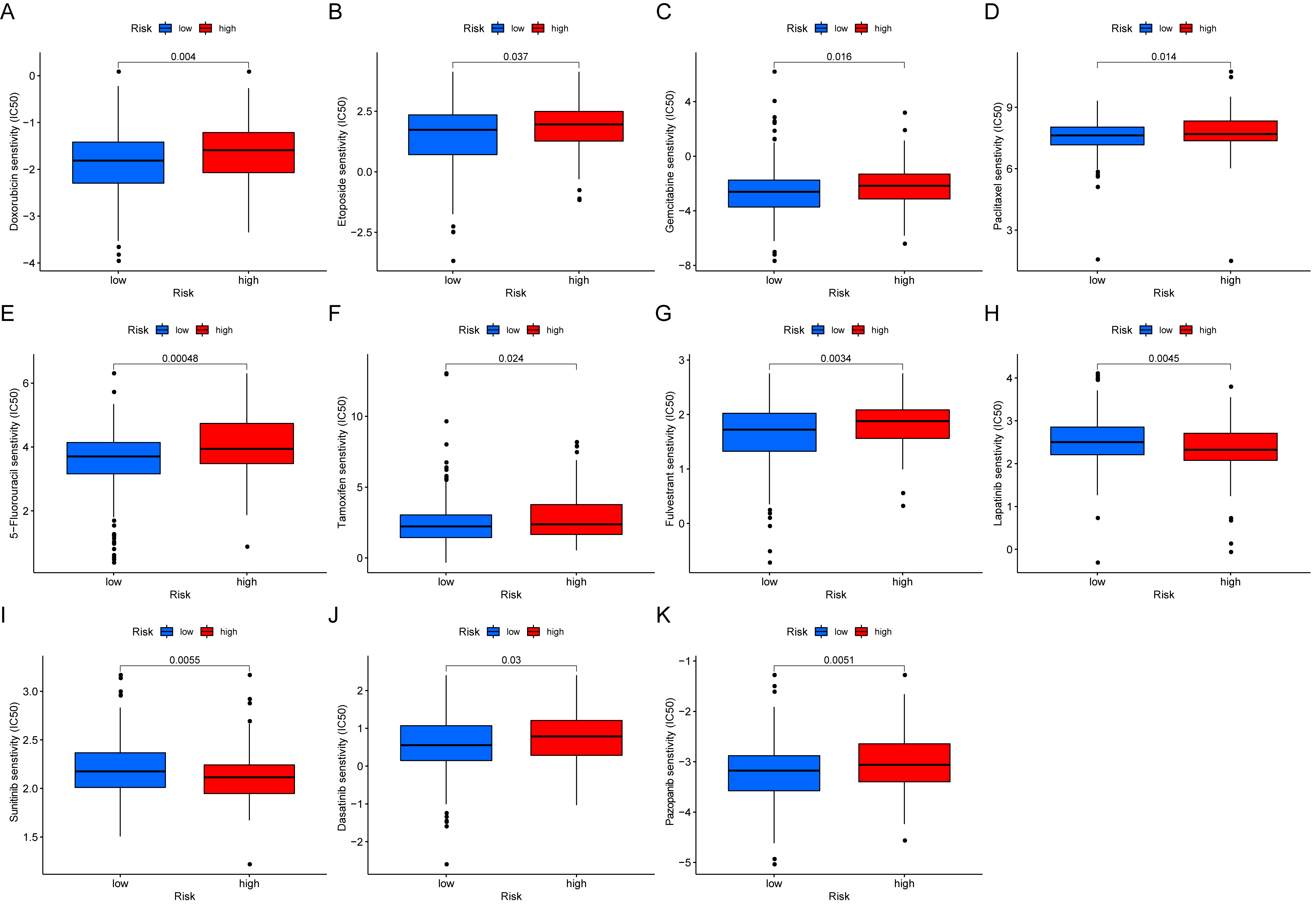

Supplement: Supplementary Figure 8 — Analysis of the association between the risk model and chemotherapeutics, endocrine therapy, and targeted therapy in the GSE86166 cohort. (A–E) The model predicting the sensitivity to chemosensitivity. It was estimated that low-risk patients had lower IC50 for chemotherapeutics of doxorubicin, etoposide, gemcitabine, paclitaxel and 5-fluorouracil. (FG) The model predicting the sensitivity to endocrine therapy. It was estimated that low-risk patients had lower IC50 of tamoxifen and fulvestrant. (H–K) The model predicting the sensitivity to targeted therapy. It was estimated that low-risk patients had lower IC50 of lapatinib, sunitinib, dasatinib and pazopanib. [file Image_8.tif]
